# A prioritization regimen of the acupuncture treatment for simple obesity: Protocol for systematic review and network meta-analysis

**DOI:** 10.1097/MD.0000000000017353

**Published:** 2019-10-25

**Authors:** Rui Zhou, Zhijie Wang, Wanwei Chen, Chushuan Huang, Chang Liu, Lixing Zhuang, Xiaoyan Xie, Haidu Hong

**Affiliations:** a Clinical College of Acupuncture, Moxibustion and rehabilitation, Guangzhou University of Chinese Medicine; b The First Clinical College, Guangzhou University of Chinese Medicine; c The First Affiliated Hospital of Guangzhou University of Chinese Medicine, Guangdong, Guangzhou, China.

**Keywords:** acupuncture, network meta-analysis, prioritization regimen, simple obesity, systematic review

## Abstract

**Background:**

Patients with simple obesity suffer from poor quality of life, as well as high risk of hypertension, diabetes, cardiovascular, and cerebrovascular accidents. Lots of Clinical trials suggested that acupuncture is beneficial for simple obesity, and it aims to gather solid evidence in order to provide reliable reference in establishing guidelines for acupuncture treatment of simple obesity in this study.

**Methods:**

Relevant databases including Cochrane Library, PubMed, Cochrane Central Register of Controlled Trials, Medline University Resource Center, Chinese Biomedical Literature Service System, and China National Knowledge Infrastructure will be retrieved from January 1950 to November 2018. Two authors will screen studies independently according to the inclusion and exclusion criteria and extract the data in a form of sheet. Quality evaluations and bias risk assessments will be performed for the methodology of included studies. Dichotomous data will be analyzed using odds ratio (OR), and continuous data using mean differences. Network meta-analysis will be conducted by using Stata 14.0. The Development and Evaluation approach will be used to rate the certainty of the evidence of estimates derived from meta-analysis. The primary outcome is body mass index (BMI), and the secondary outcomes are triglycerides, total cholesterol, low-density lipoprotein-cholesterol, effective rate, adverse effects, and recurrence rate. Trial registration number is CRD42019117387.

**Results:**

Based on current evidence, this review will rank the efficacy and safety of the various acupuncture regimen in decreasing BMI, triglycerides, total cholesterol of patients with simple obesity, and to summarize a prioritization regimen.

**Conclusion:**

This evidence may be useful for clinicians, patients, and guideline-makers to select the optimum proposal of acupuncture for the simple obesity treatment.

## 1 Introduction

Simple obesity, the most common type of obesity, is characterized by the absence of metabolic disorder and is described as a chronic disease.^[1,2]^ The etiology of simple obesity is highly complex, which involves the interaction of biological, psychological, societal, and environmental factors.^[3]^ Today, obesity has been the 2nd largest public health concern,^[4]^ and its prevalence has dramatically increased over the past 30 years.^[5]^ According to surveys,^[6]^ more than 1.9 billion adults worldwide were overweight in the year of 2016, and among these, over 650 million adults were obese. A raised of weight is significantly correlated with the risk of various diseases, such as hypertension, diabetes, cardiovascular, and cerebrovascular accidents.^[6–8]^ It is widely acknowledged that obesity kills more people than underweight does.^[9]^ Weight loss is an effective measure to reduce all kinds of adverse risks, which can be achieved by diet management and exercise, medication, and surgical treatment.^[1]^ Although diet and exercise have been playing an essential role in the weight management, their precise mode of action remains controversial.^[10]^ The management of diet and exercise can be a great challenge for some people, which require a firm will and a proper planning. Various weight loss attempts result in weight regain and poor long-term results.^[3]^ Pharmacological drugs for obesity, including sibutramine and orlistat (FDA-approved),^[11]^ could not be widely accepted due to side effects and lacking of long-term safety assurance.^[12–14]^

Traditional Chinese medicine (TCM) especially acupuncture has been an effective treatment for simple obesity.^[15]^ There is increasing scientific evidence suggests that acupuncture is beneficial for simple obesity as well as its complications.^[16–21]^ In the past decade, the number of randomized clinical trials for acupuncture has escalated gradually, and the treatment regimen of simple obesity has been increasingly diversified. Acupuncture can reduce weight through regulating glucose-inhibited neurons,^[21]^ inducing UCP1 expression, remodeling white adipose tissues to brown adipose tissue,^[22]^ and inhibiting appetite.^[23]^ After the literature searching of meta-analysis in acupuncture and related therapies for managing weight, the results showed positive. A study suggested that ear acupuncture could reduce the body weight (BM) and body mass index (BMI).^[24]^ However, the quality of acupuncture researches is variable and some meta-analyses have been produced, the guideline for acupuncture treatment of simple obesity has not yet been developed. Evidence strength of relative studies is somewhat inadequate in guiding the treatment of simple obesity. Therefore, evidence need to be collected and analyzed for the formulation of a prioritization regimen of acupuncture for simple obesity.

## 2 Data and methods

This study has been registered with PROSPERO, and registration number was CRD42019117387. It will be reported following the Preferred Reporting Items for Systematic Review.

### 2.1 Inclusion criteria

#### 2.1.1 Types of studies

There are no limitations on the language of randomized controlled trials, which baseline information were statistically balanced between each group.

#### 2.1.2 Types of participants

The criteria for participants are as follows: patients with obesity (BMI > 30^[25]^), no organic diseases, and the ability to complete the trial independently. No restrictions on gender, age, race, education level, or economy.

#### 2.1.3 Types of interventions

**Figure 1:**
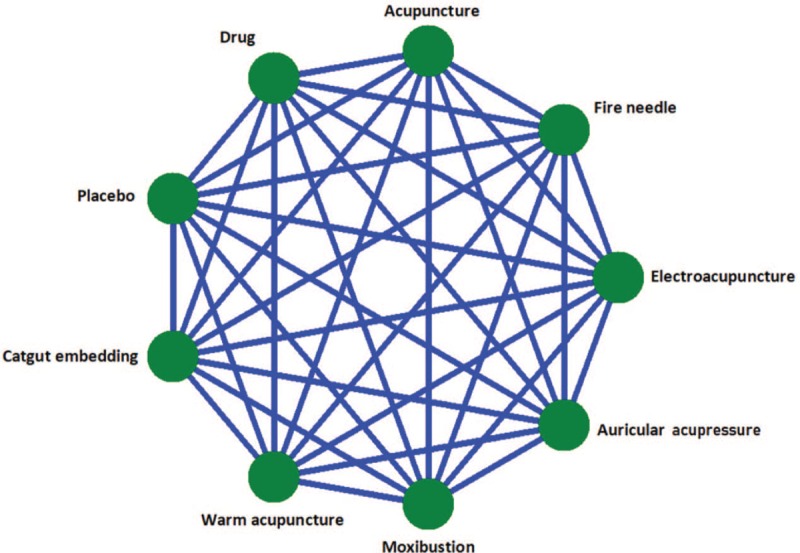
A network for all comparisons.

Interventions including acupuncture (electro-acupuncture, fire-needle, auricular needle, and warm acupuncture), moxibustion, or acupoint catgut embedding as a monotherapy will be included in this study. The interventions in control group can be exercises (ie, Taichi, Yoga, and dance), pharmaceutical drug, placebo (sham needle or sham acupoint), sham, or waitlist. Comparisons investigated are given in Figure 1.

#### 2.1.4 Types of outcomes

The primary outcome is BMI, which is an evaluation index of obesity degree, and the calculation of BMI is weight (kg) divided by the square of height (m). The secondary outcomes are including BM, body fat percentage, waist, hip, waist hip rate, total cholesterol, triglycerides, low-density lipoprotein-cholesterol, BM reducing rate, adverse effect, and recurrence rate. Total cholesterol, triglycerides, and low-density lipoprotein-cholesterol will be assessed for various vascular accidents associated with obesity. The characteristic information of included studies is shown in Table 1.

**Table 1:**
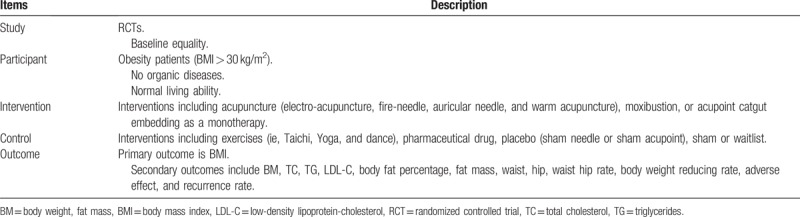
The characteristic information of included studies.

### 2.2 Studies searching

#### 2.2.1 Computer-based retrieval

We plan to collect all studies comprehensively that meet the inclusion criteria. Subject retrieval will be performed for Medline University Resource Center and China National Knowledge Infrastructure in first step to develop a retrieval strategy. According to retrieval strategy, we will search the following databases from January 1950 to November 2018: PubMed, Embase, Cochrane Central Register of Controlled Trials, Web of Science, Medline University Resource Center, Chinese Biomedical Literature Service System, China National Knowledge Infrastructure, VIP Database, and Wanfang Data. The search strategy is presented in Table 2.

**Table 2:**
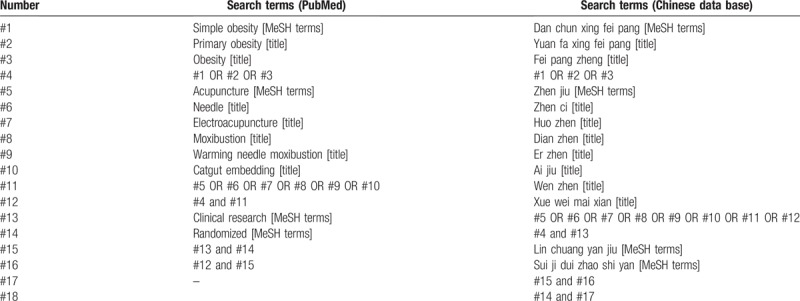
Search strategy.

#### 2.2.2 Manual retrieval

We will also search some related journals by manual including “Journal of Traditional Chinese Medicine,” “Chinese Acupuncture & Moxibustion,” “Shanghai Journal of Acupuncture and Moxibustion,” “Journal of Clinical Acupuncture and Moxibustion,” “New Chinese Medicine,” “Journal of Guangzhou University of Chinese Medicine,” and paper dissertation library in Guangzhou University of Chinese Medicine, Southern Medical University, Jinan University and Guangzhou Medical University.

### 2.3 Data collection

#### 2.3.1 Selection of studies

**Figure 2:**
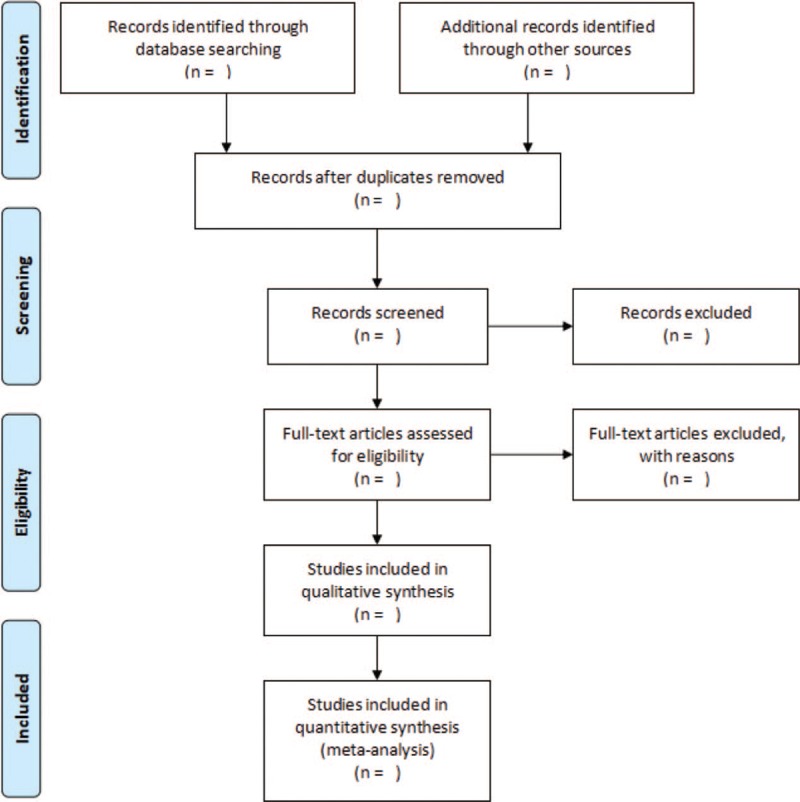
Study flow diagram.

Two authors (ZR and WZJ) will review the studies independently according to the inclusion criteria. The consistency of the result will be examined by Kappa value.^[26]^ If there are divisions in the screening process, the authors will discuss together and seek advice from a third party. Details of the selection process will be presented in the PRISMA flow chart (Fig. 2).^[27]^

#### 2.3.2 Data extraction

A data extraction sheet will be conducted for the including study, involving information on details of the first author, year of publication, study design, sample capacity, characteristics of participants, intervention, comparator, outcomes, follow-up, and adverse events.

#### 2.3.3 Assessment of quality in included studies

The standards from *Cochrane Handbook.5.1.0*^[28]^ (The Cochrane Collaboration's tool for assessing risk of bias) will be adopted to conduct quality evaluation and risk assessment of the included study methodology. The main contents include: Correctness of stochastic; Concealment of allocation; The setup of blind method; Baseline comparison; Result data integrity; Selective report results; Description of lost visit and exit; and Other sources of bias. The result of the evaluation will be input in RevMan 5.3^[29]^ to generate bias and risk proportional diagram.

### 2.4 Data analyses

#### 2.4.1 Selection of treatment effect

This review will extract data from the outcomes of each study and combine all effects of the same interventions. We will summarize the data using odds ratio (OR) with 95% confidence intervals (CIs) for dichotomous data, and using mean difference with 95% CI for continuous outcomes. If the research only provides the mean and standard deviation before and after treatment, we can use the formula, which is offered by *Cochrane Handbook.5.1.0*, to calculate the standard deviation of d-value. (If there is no correlation coefficient available in the study, we will send a mail to the writer asking for related statistics.)
 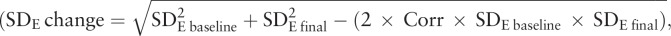


SD_E baseline_: Baseline measures; SD_E final_: Final measures; Corr: Correlation coefficient.)

#### 2.4.2 Assessment of heterogeneity

We will use χ^2^ and *I*^2^ test to estimate heterogeneity of both OR and mean difference. If *I*^2^ < 50% and *P* > .10, fixed-effect model will be used to interpret the results as low heterogeneity, whereas a random-effect model will be used.

#### 2.4.3 Evidence network diagram

Dichotomous and continuous data will be transformed into the format as shown in Table 3. An evidence network diagram which show distribution of direct and indirect evidence by Stata 14.0.^[30]^

**Table 3:**
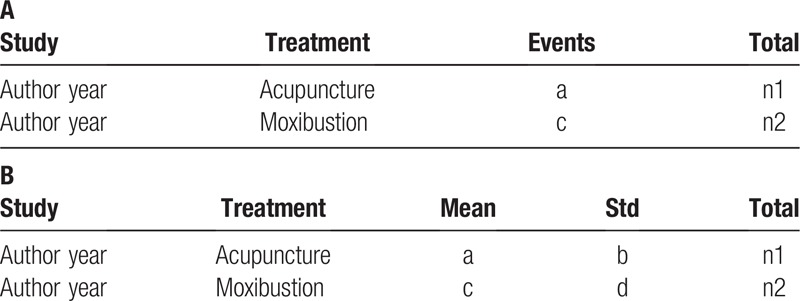
Data format (A, dichotomous data; B continuous data).

#### 2.4.4 Inconsistency examination

We plan to evaluate the consistency assumption for the entire network using the design-by-treatment interaction model.^[31]^ Triangular (closed loop formed by 3 treatments all compared with one another) loops will be assessed to confirm local inconsistency between direct and indirect evidence. Those closed loops formed by more than 3 treatments will be divided into several triangular loops. Inconsistency factor (IF) will be calculated as the absolute difference (with 95% CIs^[32]^ and a z-test) between direct and indirect estimates for each paired comparison in the loop. The IF is the logarithm of the ratio of 2 ORs from direct and indirect evidence in the loop; IF close to 0 or ratio of 2 ORs close to 1 indicates that the 2 sources are in agreement.^[33]^

#### 2.4.5 Models of consistency and inconsistency

The center group of evidence network will be chosen as the reference group. Based on the hypothesis of no heterogeneity between interventions, we will fit consistency and inconsistency model to assess the differences between 2 models’ results, and examine the inconsistency by Wald test.^[32,31]^ An inconsistency will be used to conduct network-meta analysis if inconsistency is statistically significant, whereas a consistency model will be used. We regard inconsistency as remarkable when *P* < .05 (Wald test).^[31]^

#### 2.4.6 Ranking of effects

Based on design-by-treatment interaction, we intend to calculate the surface under the cumulative ranking curve of all interventions and then sort the effects of network-meta analysis according to the cumulative ranking curve value.

#### 2.4.7 Assessment of publication biases

Publication bias may decrease the evidence intensity of systematic evaluation.^[33]^ Therefore, we plan to use a funnel plot to assess publication among included studies.

#### 2.4.8 Analysis of subgroups

If the necessary data are available, subgroup analyses will be conducted based on baseline weight status of participants (overweight [BMI: 25–29], obese [BMI: 30–39], and morbidly obese [BMI 40+]); within each class, we also plan to do a subgroup analysis by gender (male, female) or age (<20, 20–30, 30–40, 40–50, and >50). This is a qualitative synthesis and while subgroup analyses may be undertaken, it is not possible to decide the groups in advance.

#### 2.4.9 Strategy for data synthesis

We will undertake statistical analysis by using Stata 14.0 software. If the data cannot be combined in a network meta-analysis, we will provide a narrative synthesis of the findings of included studies, structured around the type of intervention, target population characteristics, and type of outcome. The synthesis of the findings will be presented along with tables and figures to aid in data presentation where appropriate.

#### 2.4.10 Confidence in cumulative evidence

We will evaluate the quality of evidence of the included studies through the Grading of Recommendations Assessment, Development and Evaluation system. The limitations of the study, inconsistencies, indirect evidence, inaccuracies, and publication bias will also be considered. Four levels of quality of evidence will be used: high, moderate, low, or very low.

## 3 Discussion

Acupuncture as a main technique of TCM has been accepted for managing many kinds of disorders in the whole world, and high interest is showing among doctors and patients.^[34]^ Studies include clinical and meta-analysis have showed the positive effect in some diseases: Luo et al reported that acupuncture has effect on primary dysmenorrhea in a network meta-analysis^[35]^; Raul et al researched that acupuncture on Hegu point could reduce pain level tested by fNIRS^[36]^; Wang et al reported that acupuncture is effective and safety for erectile dysfunction^[37]^; and Jade and Seung researched that acupuncture has effect on women's sex hormone changes.^[38]^

Research on the mechanism of acupuncture in treating simple obesity is still indefinite, while some possible functions of acupuncture have been partly elucidated^[22,39–41]^: remodel white adipose tissue, regulate central nervous system and metabolic factor, and improve insulin resistance and leptin resistance. The effects of acupuncture have been proven on animal experiments and in clinical practice. Nowadays, there are a variety of acupoint combinations in treating simple obesity. Based on the complex network analysis, it is found that^[42,43]^ frequently used meridians include the Stomach Meridian, Conception Vessel, and Bladder Meridian. Frequently used acupoints include Tianshu (ST 25), Zusanli (ST 36), and Sanyinjiao (SP 6), which play a significant role in treating simple obesity. Tianshu and Zusanli are both located on stomach meridian, and Sanyinjiao is on Spleen meridian. Those 3 acupoints always used together for controlling the function of stomach and spleen. In TCM, stomach and spleen have great relationship with dietary metabiloc, and obesity could be occurred when dietary metabiloc is disorder.

However, the guideline for acupuncture treatment of simple obesity has not yet been developed; so acupuncturists may use variable plans depend on their own experience, which may result in different effects. The evidence strength of relative studies is somewhat inadequate. This study will provide a detailed review on the efficacy and safety aspects of various acupuncture therapies for simple obesity. Solid evidence will be obtained to formulate a prioritization regimen for the treatment of simple obesity, which can be useful for clinicians, patients, and guideline-makers to select an optimum proposal of acupuncture in the simple obesity treatment.

## 4 Conclusions

This evidence may be useful for clinicians, patients, and guideline-makers to select the optimum proposal of acupuncture for the simple obesity treatment.

## Author contributions

**Conceptualization:** Rui Zhou, Zhijie Wang, Lixing Zhuang.

**Data curation:** Chushuan Huang, Chang Liu, Lixing Zhuang.

**Formal analysis:** Wanwei Chen, Xiaoyan Xie.

**Funding acquisition:** Zhijie Wang, Lixing Zhuang.

**Investigation:** Haidu Hong.

**Methodology:** Rui Zhou, Zhijie Wang.

**Project administration:** Chushuan Huang.

**Resources:** Wanwei Chen, Xiaoyan Xie.

**Software:** Rui Zhou.

**Supervision:** Chang Liu, Lixing Zhuang.

**Validation:** Lixing Zhuang.

**Visualization:** Haidu Hong.

**Writing – original draft:** Rui Zhou, Zhijie Wang, Wanwei Wang.

**Writing – review & editing:** Zhijie Wang, Lixing Zhuang.
